# Demography and sex work characteristics of female sex workers in India

**DOI:** 10.1186/1472-698X-6-5

**Published:** 2006-04-14

**Authors:** Rakhi Dandona, Lalit Dandona, G Anil Kumar, Juan Pablo Gutierrez, Sam McPherson, Fiona Samuels, Stefano M Bertozzi, the ASCI FPP Study Team

**Affiliations:** 1 Health Studies Area, Centre for Human Development, Administrative Staff College of India, Hyderabad, India; 2 Division of Health Economics and Policy, National Institute of Public Health, Cuernavaca, Mexico; 3 Research and Evaluation Unit, International HIV/AIDS Alliance, Brighton, UK

## Abstract

**Background:**

The majority of sex work in India is clandestine due to unfavorable legal environment and discrimination against female sex workers (FSWs). We report data on who these women are and when they get involved with sex work that could assist in increasing the reach of HIV prevention activities for them.

**Methods:**

Detailed documentation of demography and various aspects of sex work was done through confidential interviews of 6648 FSWs in 13 districts in the Indian state of Andhra Pradesh. The demography of FSWs was compared with that of women in the general population.

**Results:**

A total of 5010 (75.4%), 1499 (22.5%), and 139 (2.1%) street-, home-, and brothel-based FSWs, respectively, participated. Comparison with women of Andhra Pradesh revealed that the proportion of those aged 20–34 years (75.6%), belonging to scheduled caste (35.3%) and scheduled tribe (10.5%), illiterate (74.7%), and of those separated/divorced (30.7%) was higher among FSWs (p < 0.001). The FSWs engaged in sex work for >5 years were more likely to be non-street-based FSWs, illiterate, living in small urban towns, and to have started sex work between 12–15 years of age. The mean age at starting sex work (21.7 years) and gap between the first vaginal intercourse and the first sexual intercourse in exchange for money (6.6 years) was lower for FSWs in the rural areas as compared with those in large urban areas (23.9 years and 8.8 years, respectively).

**Conclusion:**

These data highlight that women struggling with illiteracy, lower social status, and less economic opportunities are especially vulnerable to being infected by HIV, as sex work may be one of the few options available to them to earn money. Recommendations for actions are made for long-term impact on reducing the numbers of women being infected by HIV in addition to the current HIV prevention efforts in India.

## Background

An estimated 4.9 million people were newly infected with HIV around the world in 2005 [1]. The proportion of women infected by HIV worldwide has grown steadily and it is estimated that half of the people currently infected by HIV are women [1,2]. Significantly higher rates of HIV infection have been documented among populations involved with sex work than in most other populations [2].

The national HIV prevalence in India is still estimated to be low (<1% in adults) but higher HIV prevalence is reported in six states, including Andhra Pradesh, where the majority of infections are acquired sexually, and the epidemic is judged to be largely linked to sex work [3]. Female sex workers (FSWs) have much higher rates of HIV infection than the pregnant women in India [3]. HIV prevalence in FSWs in Andhra Pradesh was estimated to be 16%, ranging from 8–41% in 2004 [4]. While sex work is a universal phenomenon, the majority of sex work in India is clandestine due to unfavorable legal environment [5-7], and because the Indian society discriminates against FSWs as immoral women, as in many other societies. The number of women involved with sex work is difficult to determine but it is estimated that about 1% of the adult women in India could be engaged in sex work [8], most of whom are non-brothel based sex workers [9].

Though there is a broad understanding that women in sex work tend to be young and are expected to contribute to their family income, few data are available on their demographic attributes, as many of them do not openly acknowledge that they are sex workers. For the HIV prevention programmes to increase their reach, it is important to know demographically which women are more likely to be engaged in sex work and when they get involved with sex work. In this background, we report these data for a large sample of FSWs in Andhra Pradesh in India. These data were collected as part of a baseline study for impact assessment of the Frontiers Prevention Project that aims to reduce the spread of HIV through provision of HIV prevention interventions targeting key population groups who are at higher risk of acquiring and transmitting HIV infections, including FSWs. We also make recommendations for action for HIV prevention in India based on the patterns revealed in these data.

## Methods

These data are from a baseline study designed to document the socio-demographic and sex work characteristics of FSWs and to identify issues that needed particular attention for prevention of HIV and other sexually transmitted infections. At a later stage, it is planned to compare these baseline data with a follow-up study to assess the impact of the Frontiers Prevention Project. The baseline study was approved by the Ethics Committees of the Administrative Staff College of India, Mexico's National Institute of Public Health, the International HIV/AIDS Alliance, and by the Indian Health Ministry's Screening Committee, Indian Council of Medical Research, New Delhi. Detailed methodology of this study has been reported previously [10], and the methods relevant to this paper are described below.

Forty geographic sites in 13 districts of the Telangana and Rayalseema regions of Andhra Pradesh state were identified where access to FSWs was considered feasible through non-governmental organisations having links with them. Each geographic site consisted of one or more close-by locations (cities/towns/villages). Within the 40 geographic sites, 25 locations were rural and 47 were urban of various sizes, according to the Census of India definitions [11]. The required sample size of FSWs was estimated as 6,500 to detect a significant change in high-risk sexual behaviour and in sexually transmitted infections between the baseline and follow-up studies.

Data were collected between July 2003 and April 2004. At each study location, FSWs >15 years of age were contacted and recruited through facilitators for participation in this study. Written informed consent for participation was obtained from each respondent. Standardised procedures were followed for interviewing the respondents. Training of the interviewers was done in order to address technical and ethical issues as well as to promote cultural sensitivity. One-to-one confidential interviews were done with the FSWs using a questionnaire developed by an international team with multidisciplinary background. The questionnaire was developed in English, was translated into Telugu, the local language, following which it was back-translated into English in order to ensure accurate and relevant meaning of the questions. To ensure confidentiality, the names of respondents were not recorded and hence cannot be linked to the data.

Data were entered in an LSD (Sistemas Integrales, Santiago, Chile) database, and SPSS software was used for data analyses. Three different types of FSWs participated in the study. The *street-based FSWs *who primarily solicited clients on streets (such as cinema, park, bus-stand, railway station, hotel/lodge) and provided services at hotel/lodge or a place of client's choice, *home-based FSWs *who primarily solicited clients at their own homes either directly or through a mediator and provided services at their own homes, and *brothel-based FSWs *who primarily solicited clients through an agent (such as pimp, madam) or mediator and provided services at a brothel (a place of sex work with 2 or more FSWs working under control of an agent).

Data on demography and on various aspects of when the sex work was started were analysed. The demographic characteristics of FSWs were compared with those of the women in the state of Andhra Pradesh. Data on age, caste and literacy for the women in Andhra Pradesh were obtained from the latest Census of India [11,12], and data on marital status and number of children were obtained from the National Family Health Survey-2 [13]. Duration of sex work, age at starting sex work, and gap between the first vaginal intercourse and the first sexual intercourse in exchange for money were analysed to understand when these women got involved with sex work. Univariate and bivariate analyses, where relevant, are reported.

## Results

A total of 7251 FSWs were contacted, of whom 6648 (91.7%) participated in the study. Among these, 5010 (75.4%), 1499 (22.5%), and 139 (2.1%) were street-, home-, and brothel-based, respectively.

### Demography

**Figure 1:**
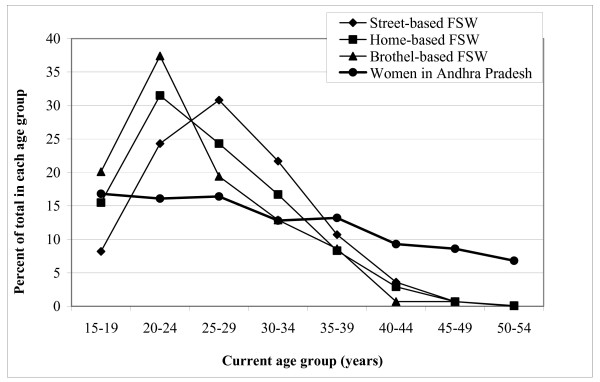
Age distribution of female sex workers and of women aged 15 to 54 years in the state of Andhra Pradesh, India [11]. For female sex workers (FSWs), the age category of 15–19 years denotes 16–19 years.

Table 1 summarises the demographic characteristics of FSWs who participated. The age range of FSWs was 16 to 54 years (standard deviation [SD] 6.33 years) with mean age of 27.8, 26.0 and 24.7 years for the street-, home- and brothel-based FSWs, respectively. On comparing the age distribution between FSWs and women aged 15 to 54 years in Andhra Pradesh (Figure 1), the proportion of those between 20–34 years was significantly higher among FSWs (75.6%) than in the women population of Andhra Pradesh (45.3%) (p < 0.0001). The proportion of brothel-based FSWs was highest in the 20–24 years age group (37.4%) followed by home-based FSWs (31.5%) as compared with the women in Andhra Pradesh (16.1%), and the proportion of street-based FSWs was highest among the 25–29 years age group (30.8%) as compared with 16.4% among the women in Andhra Pradesh.

**Table 1:** Distribution of demographic characteristics for the three types of female sex workers.

		**Type of female sex workers**				
				
**Variable**	**Variable categories**	**Street-based (N = 5010) Number (% of N)**	**Home-based (N = 1499) Number (% of N)**	**Brothel-based (N = 139) Number (% of N)**	**Total (N = 6648) Number (% of N)**	**Chi-square test for trend**
**Age group (years)**	16–19	409 (8.2)	233 (15.5)	28 (20.1)	670 (10.1)	p < 0.0001
	20–24	1215 (24.3)	472 (31.5)	52 (37.4)	1739 (26.2)	
	25–29	1541 (30.8)	365 (24.3)	27 (19.4)	1933 (29.1)	
	30–34	1087 (21.7)	251 (16.7)	18 (12.9)	1356 (20.4)	
	35–39	538 (10.7)	124 (8.3)	12 (8.6)	674 (10.1)	
	40–44	182 (3.6)	43 (2.9)	1 (0.7)	226 (3.4)	
	45–49	35 (0.7)	10 (0.7)	1 (0.7)	46 (0.7)	
	50–54	3 (0.1)	1 (0.1)	0	4 (0.1)	

Caste*	Forward	256 (5.1)	64 (4.3)	13 (9.4)	333 (5.0)	p < 0.0001
	Backward	1808 (36.1)	910 (60.7)	89 (64.0)	2807 (42.2)	
	Scheduled	1946 (38.8)	381 (25.4)	23 (16.5)	2350 (35.3)	
	Scheduled tribe	649 (13.0)	46 (3.1)	3 (2.2)	698 (10.5)	
	Not applicable	349 (7.0)	98 (6.5)	11 (7.9)	458 (6.9)	

**Education level**	Illiterate	3851 (76.9)	1017 (67.8)	98 (70.5)	4966 (74.7)	p < 0.0001
	Primary education (classes I to V)	862 (17.2)	318 (21.2)	27 (19.4)	1207 (18.2)	
	More than primary education	297 (5.9)	164 (10.9)	14 (10.1)	475 (7.1)	

**Marital status**†	Currently married	2280 (45.5)	394 (26.3)	24 (17.3)	2698 (40.6)	p < 0.0001
	Separated	1539 (30.7)	347 (23.1)	37 (26.6)	1923 (28.9)	
	Divorced	82 (1.6)	35 (2.3)	1 (0.7)	118 (1.8)	
	Widowed	642 (12.8)	139 (9.3)	11 (7.9)	792 (11.9)	
	Never married	467 (9.3)	584 (39.0)	66 (47.5)	1117 (16.8)	

**Number of living children**	None	890 (17.8)	585 (39.0)	71 (51.1)	1546 (23.3)	p < 0.0001
	1	1035 (20.7)	275 (18.3)	23 (16.5)	1333 (20.1)	
	2	1684 (33.6)	349 (23.3)	25 (18.0)	2058 (31.0)	
	>2	1401 (28.0)	290 (19.3)	20 (14.4)	1711 (25.7)	

*Not applicable for those belonging to religion other than Hindu.

†Separated: not staying with husband but not legally divorced.

The proportion of scheduled caste (35.3%) was significantly higher (p < 0.01) among the FSWs as compared with Andhra Pradesh women (16.2%) whereas the proportion of scheduled tribe was not significantly higher among the FSWs (10.5%) as compared with Andhra Pradesh women (6.6%). Illiteracy was reported by 74.7% of the FSWs as compared with 56.3% of the Andhra Pradesh women (p < 0.001).

**Figure 2:**
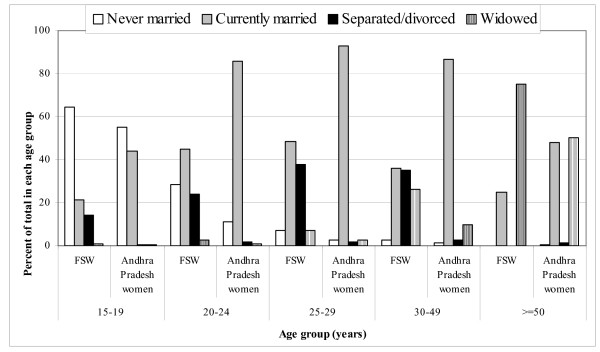
Distribution of marital status of female sex workers (FSWs) and of women in the state of Andhra Pradesh, India [13]. The age category of 15–19 years denotes 16–19 years for FSWs.

The proportion of those never married was much higher among the non-street-based as compared with the street-based FSWs (p < 0.001) as shown in Table 1. Overall, the proportion of those currently married was much lower (40.6%) and of those separated/divorced much higher (30.7%) among FSWs as compared with Andhra Pradesh women for whom these proportions were 71.4% and 1.4%, respectively (p < 0.001) (Figure 2). The proportion of those currently married was more than 80% among the women in Andhra Pradesh and less than 50% among FSWs in the 20–24, 25–29 and 30–49 years age group, respectively. For the age group 16–49 years, the proportion of those widowed was 11.9% and 5% for FSWs and Andhra Pradesh women, respectively.

A total of 5102 (76.7%) FSWs reported having at least one living child, of which 152 (3%) were never married. Among the FSWs who were aged 16–24 years and were never married, the proportion of those having children was 2.2%, 14.7% and 5.3% among the street-, home- and brothel-based FSWs, respectively. Of all the FSWs who had children, 4169 (81.7%) reported that all their children were staying with them and 639 (12.5%) reported that at least one but not all of their children were staying with them. Irrespective of the marital status, the overall mean number of living children for FSWs was 2.2 (range 1 to 8; SD 1.03) and that for Andhra Pradesh women was 2.0 (Table 2). The mean number of children was higher for FSWs in the age group 16–24 years but was lower in the other age groups as compared with the respective age groups for women in Andhra Pradesh (Table 2), and this trend was also seen when only currently married FSWs and currently married women in Andhra Pradesh were compared.

**Table 2:** The mean number of living children for female sex workers and for women of the state of Andhra Pradesh, India.

**Age group (years)**	All women*†		**Currently married women**†	
	
	**FSW‡(6648)**	**Andhra Pradesh‡ (4686)**	**FSW‡ (2698)**	**Andhra Pradesh§ (3695)**
15–19¶	1.26	0.27	1.23	0.60
20–24	1.58	1.32	1.62	1.53
25–29	2.04	2.22	2.14	2.33
30–34	2.60	2.66	2.84	2.76
35–39	2.89	3.04	3.05	3.18
40–44	3.14	3.15	3.28	3.29
45–49	2.88	3.54	2.73	3.77
50–54#	2.67	-	0.00	-
TOTAL	2.22	2.03	2.22	2.38

*Irrespective of marital status.

†Chi-square test for trend is not significant.

‡FSW: female sex worker.

§Data from the National Family Health Survey – 2 [13]; data not available for women aged 50–54 years.

¶16–19 years age category for FSWs.

#Only 1 FSW in this age group was currently married who reported no children.

### Sex work

#### Duration of sex work

Table 3 summarises select characteristics of FSWs based on the duration of sex work. Considering all FSWs together, the mean duration of sex work was 4.21 years (95% confidence interval: 4.08–4.34 years). A total of 1113 (16.7%) FSWs reported that they were in sex work for 1 year or less at the time of interview. The proportion of those who were in sex work for >5 years was higher among the non-street-based FSWs, those who were illiterate, those living in small urban towns, and those who started sex work early at the age of 12–15 years (p < 0.001).

**Table 3:** Distribution of select characteristics of female sex workers (FSWs) for the different durations of sex work.

			**Duration of sex work (years)**			
			
**Variable**	**Variable categories**	**Total (6648)**	**>5 (N = 1499) (% of total) [% of N]**	**>3–5 (N = 1617) (% of total) [% of N]**	**>1–3 (N = 2419) (% of total) [% of N]**	**≤1 (N = 1113) (% of total) [% of N]**
Type of FSW*	Street-based	5010	946 (18.9) [63.1]	1196 (23.9) [74.0]	1952 (39.0) [80.7]	916 (18.3) [82.3]
	Home-based	1499	514 (34.3) [34.3]	382 (25.5) [23.6]	429 (28.6) [17.7]	174 (11.6) [15.6]
	Brothel-based	139	39 (28.1) [2.6]	39 (2.4) [2.4]	38 (27.3) [1.6]	23 (16.5) [2.1]
Literacy *	Illiterate	4966	1198 (24.1) [79.9]	1244 (25.1) [76.9]	1198 (24.1) [71.4]	798 (16.1) [71.7]
	Literate	1682	301 (17.9) [20.1]	373 (22.2) [23.1]	301 (17.9) [28.6]	315 (18.7) [28.3]
Rural-urban area from where FSW was sampled*†	Rural	1447	334 (23.1) [22.3]	352 (24.3) [21.8]	506 (35.0) [20.9]	255 (17.6) [22.9]
	Urban small	749	225 (30.0) [15.0]	196 (26.2) [12.1]	249 (33.2) [10.3]	79 (10.5) [7.1]
	Urban medium	2838	606 (21.4) [40.4]	695 (24.5) [43.0]	1085 (38.2) [44.9]	452 (15.9) [40.6]
	Urban large	1614	334 (20.7) [22.3]	374 (23.2) [23.1]	579 (35.9) [23.9]	327 (20.3) [29.4]
Age at starting sex work (years)*‡	12–15	580	278 (47.9) [18.5]	151 (26.0) [9.3]	145 (25.0) [6.0]	6 (1.0) [0.5]
	16–19	1392	291 (20.9) [19.4]	281 (20.2) [17.4]	528 (37.9) [21.8]	292 (21.0) [26.2]
	20–24	2017	381 (18.9) [25.4]	434 (21.5) [26.9]	776 (38.5) [32.1]	426 (21.1) [38.3]
	25–29	1763	355 (20.1) [23.7]	486 (27.6) [30.1]	647 (36.7) [26.7]	275 (15.6) [24.7]
	30–34	692	161 (23.3) [10.7]	205 (29.6) [12.7]	238 (34.4) [9.8]	88 (12.7) [7.9]
	> = 35	203	33 (16.3) [2.2]	59 (29.1) [3.7]	85 (41.9) [3.5]	26 (12.8) [2.3]
Gap between first vaginal intercourse and sexual intercourse in exchange for money (years)*‡§	None	922	355 (38.5) [23.7]	238 (25.8) [14.7]	269 (29.2) [11.1]	60 (6.5) [5.4]
	1–2	481	116 (24.1) [7.7]	109 (22.7) [6.7]	177 (36.8) [7.3]	79 (16.4) [7.1]
	3–5	1062	186 (17.5) [12.4]	201 (18.9) [12.4]	411 (38.7) [17.0]	264 (24.9) [23.7]
	6–10	2102	402 (19.1) [26.8]	485 (23.1) [30.0]	810 (38.5) [33.5]	405 (19.3) [36.4]
	11–15	1393	312 (22.4) [20.8]	387 (27.8) [24.0]	489 (35.1) [20.2]	205 (14.7) [18.4]
	>15	686	128 (18.7) [8.5]	195 (28.4) [12.1]	263 (38.3) [10.9]	100 (14.6) [9.0]

*p < 0.001, Chi-square test.

†Urban small were towns with population <50000; urban medium were towns/cities with population 50001 – 200000; urban large were cities with population more than 200000; this classification was done based on Census of India data for each sub-site.

‡Age at starting sex work not available for 1 FSW, and gap between first vaginal intercourse and starting sex work not available for 2 FSWs.

§None: Age at the first vaginal intercourse and at starting sex work is same.

The proportion of FSWs who had done sex work for 10 months or more during the 12 months preceding the interviews was 80.2% and 87.3% among the street- and non-street-based FSWs, respectively (p < 0.001). Considering the 5535 FSWs who had been in sex work for >1 year, the mean duration of sex work was 4.04, 5.36 and 5.01 years for the street-, home- and brothel-based FSWs, respectively

#### Age at first vaginal intercourse and at starting sex work

**Figure 3:**
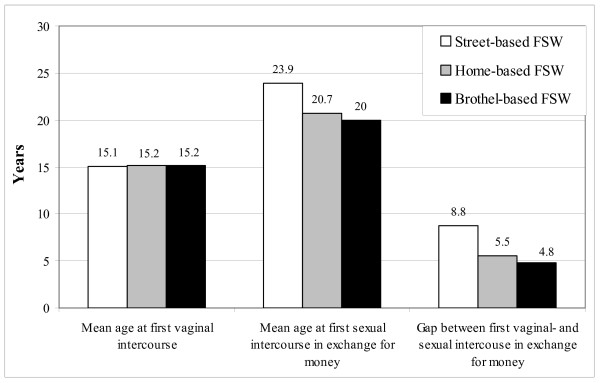
Distribution of age at first vaginal intercourse and at first sexual intercourse in exchange for money, and the gap between the two for the three types of female sex workers (FSWs).

Considering all FSWs together, the mean age at first vaginal intercourse was 15.1 years (range 10 to 30 years; SD 1.69 years) and at starting sex work was 23.1 years (range 12 to 47 years; SD 5.65 years). The mean age at first vaginal intercourse was similar for the different types of FSWs (15.1–15.2 years) (Figure 3). The mean (median, range) age at starting sex work was 23.0 (22.0, 12 to 46), 23.4 (23.0, 13 to 46), 23.6 (24.0, 12 to 43) and 22.1 (22.0, 12 to 47) years for FSWs who were in sex work for ≤1 year, >1–3, >3–5, and >5 years, respectively. The mean (median) age at starting sex work was lower for FSWs in rural areas [21.7 (21.0) years] as compared with those in small-sized urban areas [22.3 (22.0) years], medium-sized urban areas [23.6 (24.0) years] and large urban areas [23.9 (24.0) years], however, the difference was not significant.

The age at starting sex work, and the gap between the first vaginal intercourse and first sexual intercourse in exchange of money, were lower among the non-street-based as compared with the street-based FSWs (p < 0.001) (Figure 3). The mean gap between the first vaginal intercourse and the first sexual intercourse in exchange for money was the least for FSWs in rural areas (6.6 years) and the most among those in the large urban areas (8.8 years). Those who started sex work between 12–15 years of age, were never married, literate, non-street-based FSW, belonged to backward caste, and lived in rural areas were more likely to have had no gap between the first vaginal intercourse and the first sexual intercourse in exchange for money (Table 4). Those who started sex work at the age of >19 years, were ever married, illiterate, street-based FSW, belonged to caste other than backward, and lived in medium and large urban towns were more likely to be with a gap of >5 years between the first vaginal intercourse and the first sexual intercourse in exchange for money (p < 0.001).

**Table 4:** Distribution of select variables for the gap between first vaginal intercourse and the first sexual intercourse in exchange for money for female sex workers (FSWs).

			Gap between first vaginal intercourse and first sexual intercourse in exchange for money (years)*					
			
**Variable**	**Variable categories**	**Total (6646)**	**None (N = 922) (% of total) [% of N]**	**>0–2 (N = 481) (% of total) [% of N]**	**>2–5 (N = 1062) (% of total) [% of N]**	**>5–10 (N = 2102) (% of total) [% of N]**	**>10–15 (N = 1393) (% of total) [% of N]**	**>15 (N = 686) (% of total) [% of N]**
**Age at starting sex work (years)**†	12–15	580	481 (82.9) [52.2]	98 (16.9) [20.4]	1 (0.2) [0.1]	0	0	0
	16–19	1391	414 (29.8) [44.9]	348 (25.0) [72.3]	589 (42.3) [55.5]	40 (2.9) [1.9]	0	0
	20–24	2017	26 (1.3) [2.8]	34 (1.7) [7.1]	452 (22.4) [42.6]	1432 (71.0) [68.1]	73 (3.6) [5.2]	0
	25–29	1763	0	1 (0.1) [0.2]	19 (1.1) [1.8]	613 (34.8) [29.2]	1087 (61.7) [78.0]	43 (2.4) [6.3]
	30–34	692	1 (0.1) [0.1]	0	1 (0.1) [0.1]	17 (2.5) [0.8]	228 (32.9) [16.4]	445 (64.3) [64.9]
	> = 35	203	0	0	0	0	5 (2.5) [0.4]	198 (97.5) [28.9]

**Type of FSW**†	Street-based	5010	364 (7.3) [39.5]	316 (6.3) [65.7]	846 (16.9) [79.7]	1731 (34.6) [82.4]	1179 (23.5) [84.6]	574 (11.5) [83.7]
	Home-based	1497	508 (33.9) [55.1]	149 (10.0) [31.0]	197 (13.2) [18.5]	337 (22.5) [16.0]	199 (13.3) [14.3]	107 (7.1) [15.6]
	Brothel-based	139	50 (36.0) [5.4]	16 (11.5) [3.3]	19 (13.7) [1.8]	34 (24.5) [1.6]	15 (10.8) [1.1]	5 (3.6) [0.7]

**Marital status**†‡	Never married	1116	816 (73.1) [88.5]	189 (16.9) [39.3]	82 (7.3) [7.7]	24 (2.2) [1.1]	4 (0.4) [0.3]	1 (0.1) [0.1]
	Currently married	2697	58 (2.2) [6.3]	170 (6.3) [35.3]	550 (20.4) [51.8]	1083 (40.2) [51.5]	578 (21.4) [41.5]	258 (9.6) [37.6]
	Others	2833	48 (1.7) [5.2]	122 (4.3) [25.4]	430 (15.2) [40.5]	995 (35.1) [47.3]	811 (258.6) [58.2]	427 (15.1) [62.2]

**Caste**†§	Forward	333	33 (9.9) [3.6]	25 (7.5) [5.2]	59 (17.7) [5.6]	104 (31.2) [4.9]	69 (20.7) [5.0]	43 (12.9) [6.3]
	Backward	2805	586 (20.9) [63.6]	242 (8.6) [50.3]	439 (15.7) [41.3]	812 (28.9) [38.6]	510 (18.2) [36.6]	216 (7.7) [31.5]
	Scheduled	2350	209 (8.9) [22.7]	131 (5.6) [27.2]	388 (16.5) [36.5]	799 (34.0) [38.0]	566 (24.1) [40.6]	257 (10.9) [37.5]
	Scheduled tribe	698	37 (5.3) [4.0]	61 (8.7) [12.7]	120 (17.2) [11.3]	237 (34.0) [11.3]	134 (19.2) [9.6]	109 (15.6) [15.9]
	Not applicable	458	55 (12.0) [6.0]	22 (4.8) [4.6]	56 (12.2) [5.3]	150 (32.8) [7.1]	114 (24.9) [8.2]	61 (13.3) [8.9]

**Education level**†	Illiterate	4965	576 (11.6) [62.5]	322 (6.5) [66.9]	765 (15.4) [72.0]	1589 (32.0) [75.6]	1101 (22.2) [79.0]	612 (12.3) [89.2]
	Primary education (classes I to V)	1206	242 (20.1) [26.2]	104 (8.6) [21.6]	204 (16.9) [19.2]	382 (31.7) [18.2]	215 (17.8) [15.4]	59 (4.9) [8.6]
	More than primary education	475	104 (21.9) [11.3]	55 (11.6) [11.4]	93 (19.6) [8.8]	131 (27.6) [6.2]	77 (16.2) [5.5]	15 (3.2) [2.2]

**Rural-urban area from where FSW was sampled**†¶	Rural	1446	348 (24.1) [37.7]	142 (9.8) [29.5]	224 (15.5) [21.1]	362 (25.0) [17.2]	228 (15.8) [16.4]	142 (9.8) [20.7]
	Urban small	748	131 (17.5) [14.2]	57 (7.6) [11.9]	143 (19.1) [13.5]	215 (28.7) [10.2]	142 (19.0) [10.2]	60 (8.0) [8.7]
	Urban medium	2838	299 (10.5) [32.4]	181 (6.4) [37.6]	460 (16.2) [43.3]	952 (33.5) [45.3]	665 (23.4) [47.7]	281 (9.9) [41.0]
	Urban large	1614	144 (8.9) [15.6]	101 (6.3) [21.0]	235 (14.6) [22.1]	573 (35.5) [27.3]	358 (22.2) [25.7]	203 (12.6) [29.6]

*None denotes that the age at first vaginal intercourse and at starting sex work was same.

†p < 0.001, Chi-square test; data not available for 2 FSWs.

‡Others include separated/divorced/widowed.

§Not applicable for those belonging to religion other than Hindu.

¶Urban small were towns with population <50000; urban medium were towns/cities with population 50001 – 200000; urban large were cities with population more than 200000; this classification was done based on Census of India data for each sub-site.

## Discussion

We have reported data on demography and sex work for a large sample of the different types of FSWs from urban and rural parts of the state of Andhra Pradesh, which is one of the high HIV prevalence states in India [3,4] As the FSWs who participated in this study were recruited through FSW facilitators, these FSWs may not be representative of all FSWs, thereby, suggesting a bias towards those who are better connected with their peers, and hence the results should be interpreted within this limitation.

### Demography

Eighty six percent of all the FSWs who participated in this study were between 15 to 34 years of age. The proportion of FSWs aged 20–34 years was 1.7 times more than that of the women in the same group in Andhra Pradesh. The brothel- and home-based FSWs tended to be younger as compared with the street-based FSWs. The brothel houses usually employ young women and girls, as these women/girls can be kept in their services for a longer duration. The home-based FSWs in our study also included traditional sex workers belonging to Dommuri community. These are typically young girls who are in sex work because of the tradition that the elder girl of the family is required to economically support the household through sex work. The focus groups conducted with them revealed that they start sex work immediately after attaining puberty, and the clients are charged Indian Rupees 3000 to 8000 (US$ 69–184) for the first sex with these girls.

The proportion of those belonging to scheduled caste and scheduled tribe was higher among FSWs as compared with the women of Andhra Pradesh. The Indian population is sub-divided into four castes – forward caste, backward caste, scheduled caste and scheduled tribe by virtue of birth in the family of a particular caste. These castes are also a surrogate measure for the socioeconomic status of the people as those belonging to forward caste have the highest socioeconomic status and the scheduled tribes have the lowest. Separate hamlets for people belonging to scheduled caste and scheduled tribe are not uncommon in Indian villages because those belonging to the forward caste consider them untouchables. In the background of limited economic and social opportunities available to people belonging to scheduled caste and scheduled tribe, it is not surprising that these women have a higher representation in sex work as compared with the women belonging to the forward and backward castes.

Illiteracy, again, is associated with less economic opportunities. The literacy rate in India among men is 75.3% and is 53.7% among women [12], and 41.5% of the illiterate men and 30% of the illiterate women were involved in some economic activity [14]. Traditionally in the Indian society, women after marriage are expected to take care of the household, children, and assist with the work of the men of the household (for example – work in agriculture) but are not encouraged to work outside their household for generating income as that responsibility lies with the men. However, with increasing poverty and decreasing economic opportunities, married women are increasingly seeking work outside their households to generate income. And, it is likely that the earning potential in sex work for the poor and illiterate women is larger to what they could earn through other types of work.

As expected, the proportion of single women was higher among the FSWs as compared with the women of Andhra Pradesh. These single women included those never married, those separated or divorced from their husbands, and those widowed. The proportion of never married FSWs was higher among the brothel- and home-based FSWs as compared with the street-based FSWs because the former tended to be younger as mentioned previously. Women who are separated/divorced from their husbands or are widowed have limited rights, and economic independence [15]. In addition, if they are illiterate, they are likely to have even fewer labour market opportunities other than sex work.

The overall number of mean children was not very different between the FSWs and the women of Andhra Pradesh. However, FSWs between 16 to 24 years of age had a higher number of children as compared with those in the same age group in Andhra Pradesh. A greater number of children may be attributed to the nature of work that they are involved with. On the other hand, it may also be that they are drawn into sex work to seek greater earnings because of the need to support a larger number of children. Focus group discussions with the home-based FSWs revealed that it was not uncommon for the young unmarried traditional sex workers to have a child of the man whom they liked. Interestingly, these women preferred to bear child of men belonging to the forward caste to increase the chances of the child having fair complexion, which is considered desirable in the Indian society.

### Sex work

The non-street-based FSWs, illiterate, living in small urban towns, and who started sex work at the age of 12–15 years were more likely to be in sex work for more than 5 years. The mean duration of sex work was the least for street-based FSWs. This finding reinforces the fact that the turnover of FSWs is relatively more among the street-based as compared with the non-street-based FSWs. The street-based FSWs are the *informal *(unorganized) sex workers as compared with non-street-based FSWs who are more organized. In our study, the proportion of FSWs who had worked as sex workers for the majority of the 12 months were higher among the non-street-based as compared with street-based FSWs.

The mean age at starting sex work for FSWs from rural areas was lower to those in the large urban areas. Related to this, the gap between the first vaginal intercourse and that in exchange for money was also the least for FSWs in rural areas and the most for those in large urban areas. This finding highlights that the economic opportunities for women in rural areas are relatively less than those in the urban areas, and hence the former get into sex work earlier than the latter.

Interestingly, the mean age at first vaginal intercourse was similar for the different types of FSWs but the gap between the first vaginal intercourse and that in exchange for money was less for non-street-based FSWs. The proportion of FSWs having a gap of more than 5 years between first vaginal intercourse and that in exchange for money was higher among the street-based FSWs as compared with the non-street based FSWs, thereby, highlighting that the former get involved with sex work later and hence are more likely to be adult women as compared with the latter. As the street-based FSWs were also more likely to be currently married/separated/divorced as compared with the non-street-based FSWs, it can be speculated that their impetus to get into sex work is to contribute to family income and they could, in some cases, be the sole supporters of their families.

### Recommendations for action

In the background of increasing numbers of women infected with HIV, there is a need to address the specific factors that contribute to women's vulnerability and risk. This increased risk of HIV in women is also a reflection of gender inequalities [2]. Recommendations for action are made within the context of HIV/AIDS prevention progamme in India based on the findings of our study.

#### Effective linkages for empowering women

The National AIDS Control Organization (NACO) is responsible for the national HIV/AIDS programme in India. NACO envisions that targeted interventions among the high-risk populations that include behaviour change, health care, treatment of sexually-transmitted diseases, provision of condoms, and creating an enabling environment for behaviour change are an effective way of reducing HIV/AIDS [16]. On the other hand, it is now well established that HIV/AIDS is a broad development issue and not merely a disease and that it is not just a problem of individual behaviour but is influenced by social, economic, cultural marginalisation and gender inequality [2]. Extrapolations of data from our study suggest that many women enter sex work due to economic reasons. These economic reasons, in turn, are associated with illiteracy, lower status in the society, limited economic options for women, and separation/divorce from husband or death of husband in case of married women. Therefore, it is difficult to envisage a decrease in the numbers of women affected with HIV/AIDS unless the basic factors that contribute to women's vulnerability and risk of HIV are addressed. NACO has many partnerships and collaborations for HIV/AIDS prevention programmes [17], including that with the Department of Family Welfare (DoFW) [18] that is responsible for provision of maternal and child care services and population control [19]. However, no clear linkages are established with the Department of Women and Child Development (DoWCD), the mandate of which is to empower women both economically and socially to become equal partners in national development along with men through provision of welfare and support services, training for employment and income generation, awareness generation and gender sensitization [20]. It would seem prudent to have effective collaboration between NACO and DoWCD for a holistic long-term approach to deal with HIV/AIDS, with special focus on women who are aged 21–34 years, illiterate, and belonging to scheduled caste and schedule tribe.

#### Involvement of men

These data indicate that the street-based FSWs are more likely to be currently married and that their turnover is higher as compared with non-street-based FSWs. Therefore, it is likely that street-based FSWs are those women who get into sex work as and when needed. We have previously reported from the same FSW population that 47.2% of FSWs had reported non-use of condom with at least one of her last three clients, and that the street-based FSWs were at a higher risk of HIV infection because they used condom less often with the clients as compared with the home- and brothel-based FSWs [10]. FSWs also reported almost negligible use of condom with their regular partners [10]. Condom use by married couples is low in India (0.7% by currently married women in Andhra Pradesh) because condoms are seen as a contraceptive measure, and the majority of the contraception need is met by female/male sterilization [13]. Several myths, misperceptions and fears that hinder access to and use of condoms between married couples have been identified [21]. These would have to be addressed effectively to promote use of condom with regular partners in addition to the clients. Within the Indian societal context, promoting condom with regular partners is not possible without the involvement of men.

#### Legal context of sex work

The legal status of sex work has significant bearing on the effectiveness of HIV/AIDS programmes targeting sex workers. In India, the legal context of sex work is quite complex, and FSWs are held by police under the *Immoral Traffic Prevention Act *that deals with human trafficking [5]. There are indications that the present laws tend to penalize FSWs and not the exploiters or clients even though prostitution by itself is not a crime under this Act [6,22]. More often that not, such legal structure results in making the already invisible sex worker populations more inaccessible to HIV prevention programmes, decrease the availability of health care services for them, and increase the risk of violence [6]. Therefore, attempts have to be made to change the unfavourable legal environment for these women to increase their access to the wide range of support services, including HIV prevention. Effective collaboration between NACO, law-makers and implementers, and DoWCD are needed to achieve such a change in the environment.

The above actions will have to be carried out simultaneously with the current HIV interventions in the country in order to have a long-term impact on HIV/AIDS and on women in India.

## Conclusion

These data highlight that women struggling against adverse conditions such as illiteracy, lower status in society, and less economic opportunities are especially vulnerable to being infected by HIV, as sex work may be one of the few economic options available for these women. In addition to the current HIV prevention efforts in India, attempts are needed to form effective linkages between the HIV prevention programmes and those that empower women, increase the involvement of men in HIV prevention activities, and to address the unfavourable legal environment for sex work in order to have a long-term impact on reducing the numbers of HIV infections in India.

## Competing interests

The author(s) declare that they have no competing interests.

## Authors' contributions

RD contributed to the study design, data collection, data analysis and interpretation, and drafted the manuscript. LD contributed to the study design, data collection, and data analysis and interpretation. GAK contributed to the data management, analysis and interpretation. JPG contributed to the study design, data interpretation, and coordination. SM, FS and SMB contributed to the study design, data interpretation and coordination. All named authors read, commented on and approved the final version of the manuscript. The ASCI FPP Study Team contributed to the planning of the study logistics, data collection and interpretation, and the members of this Team other than the named authors include (in alphabetical order): G Md Mushtaq Ahmed, Md Akbar, Md Abdul Ameer, Ch Arjun, N Arjun, M Sai Baba, C Satish Babu, J Kishore Babu, I Balasubrahmanyam, V S Udaya Bhaskar, T Gangadhar, P Gopal, Lavanya Gotety, Shaik Omar Hussain, V Indira, S Krishna, P Kiran Kumar, Ch Sri Jaya Lakshmi, T Uma Maheshwar, P Chandra Mouli, S Radhakrishnan, K Raghu, S P Ramgopal, A Srinivas Rao, A Srinivasa Rao, K Hanumantha Rao, N Ananda Rao, P Venkateswara Rao, Parsa V R Rao, D Ravinder, A Srinivas Reddy, G Brahmananda Reddy, S Krishna Reddy, G Uma Sankar, A Satyam, Y S Sivan, P V Sridhar.

## Pre-publication history

The pre-publication history for this paper can be accessed here:


